# Racial and Ethnic Disparity in Preoperative Chemosensitivity and Survival in Patients With Early-Stage Breast Cancer

**DOI:** 10.1001/jamanetworkopen.2023.44517

**Published:** 2023-11-22

**Authors:** Arya Mariam Roy, Archit Patel, Kayla Catalfamo, Kristopher Attwood, Thaer Khoury, Song Yao, Shipra Gandhi

**Affiliations:** 1 Department of Hematology and Oncology, Roswell Park Comprehensive Cancer Center, Buffalo, New York; 2 Department of Biostatistics, Roswell Park Comprehensive Cancer Center, Buffalo, New York; 3 Department of Pathology, Roswell Park Comprehensive Cancer Center, Buffalo, New York; 4 Department of Cancer Prevention & Control, Roswell Park Comprehensive Cancer Center, Buffalo, New York

## Abstract

**Importance:**

It remains unclear what survival benefit is associated with preoperative chemosensitivity after receiving neoadjuvant chemotherapy (NACT) among patients with resectable breast cancer from diverse racial and ethnic backgrounds.

**Objective:**

To investigate racial and ethnic disparities in chemosensitivity and association with survival in patients with early-stage breast cancer.

**Design, Setting, and Participants:**

This retrospective cohort study queried data from the National Cancer Database (NCDB) between calendar years 2010 and 2018. Participants included patients with breast cancer with clinical stage I to III disease treated with NACT. Preoperative chemosensitivity was defined as very sensitive (ypT0N0), sensitive (pathologic TNM stage less than clinical stage, excluding ypT0N0), and refractory (pathologic stage greater than or equal to clinical stage). Data were analyzed in November 2022.

**Exposure:**

Receipt of NACT and clinicopathologic and treatment factors contributing to racial and ethnic disparities in survival.

**Main Outcomes and Measures:**

Overall survival of patients from diverse racial and ethnic backgrounds who received NACT.

**Results:**

This study included 103 605 patients (median age, 53 [IQR, 44-62] years, 99.5% [n = 103 060] women, and 68.7% [n = 71 203] White race). Among them, breast cancer was refractory in 43.2% (n = 44 796), sensitive in 34.4% (n = 35 638), and very sensitive in 22.4% (n = 23 171) of patients. In the hormone receptor–positive *ERBB2* negative (formerly *HER2* negative) group, patients had more refractory disease regardless of race or ethnicity (all races and ethnicities refractory: 54%-59%; *P* < .001). Among *ERBB2* positive disease, Black patients had a lower percentage of very sensitive disease (32% vs 37%-40%; *P* < .001) and among triple-negative breast cancer, more refractory disease was seen among Black patients compared with other races and ethnicities (38% vs 30%-35%; *P* < .001). In refractory (hazard ratio [HR], 1.53; 95% CI, 1.47-1.60; *P* < .001) and sensitive (HR, 1.25; 95% CI, 1.17-1.33; *P* < .001) disease, Black patients had a higher mortality risk compared with White patients in the overall cohort. Asian patients had a lower mortality risk compared with White patients in refractory (HR, 0.71; 95% CI, 0.63-0.80; *P* < .001), sensitive (HR, 0.58; 95% CI, 0.49-0.69; *P* < .001), and very sensitive (HR, 0.60; 95% CI, 0.43-0.82; *P* < .001) disease groups in the overall cohort.

**Conclusions and Relevance:**

In this cohort study, Black patients had a higher mortality risk compared with White patients among those with residual disease after NACT. This highlights the need for personalized treatment strategies for Black patients to help them attain pathologic complete response.

## Key Points

**Question:**

Is there racial and ethnic disparity in preoperative chemosensitivity in early-stage breast cancer?

**Findings:**

In this cohort study of 103 605 patients with early-stage breast cancer who received neoadjuvant chemotherapy, Black patients had more refractory disease in *ERBB2*-positive and triple-negative breast cancer. Black patients had significantly inferior survival rates compared with other races and ethnicities across all breast cancer subtypes, and this disparity was pronounced among those with sensitive and refractory preoperative chemosensitivity.

**Meaning:**

These findings suggest the presence of racial and ethnic disparity in preoperative chemosensitivity among patients with early-stage breast cancer and that there is a need for personalized treatment options for Black patients.

## Teaser

This cohort study examines the association of chemosensitivity with survival by race and ethnicity in patients with early-stage breast cancer.

## Introduction

Breast cancer is the most common cancer among women and the second leading cause of cancer-related deaths in the US.^1^ Neoadjuvant chemotherapy (NACT) is the current standard of care for locally advanced breast cancer.^2^ Studies have shown that patients who achieve pathologic complete response (pCR) after receiving NACT have substantially better overall survival (OS) and event-free survival, with thereby a better prognosis, compared with those who have residual disease.^3,4^ This result was more prominently observed in *ERBB2*-positive (*ERBB2*^+^) (formerly *HER2*^+^) breast cancer and triple-negative breast cancer (TNBC).^4,5^

Despite advances in screening, diagnosis, and treatment of breast cancer and overall improvement in survival, racial disparities persist in breast cancer outcomes. Black women are more likely to be diagnosed with aggressive subtypes of breast cancer and have substantially higher all-cause and breast cancer–specific mortality compared with White women.^6^ Age-adjusted mortality among Black patients is still higher compared with White patients, even when cancer treatments are similar. Rates of pCR have increased over time, especially among *ERBB2*^+^ tumors given the addition of dual anti-*ERBB2* treatments in the neoadjuvant therapy. Despite improvement in pCR rates with novel therapies, there exist racial and ethnic disparities in breast cancer outcomes. Black women continue to experience lower pCR for *ERBB2*^+^ and TNBC compared with other races and ethnicities.^7^

In this study, we investigated the clinical outcomes of patients with early-stage breast cancer based on their preoperative chemosensitivity to NACT, using data from the National Cancer Database (NCDB). We aimed to analyze the racial and ethnic disparities in the preoperative chemosensitivity and compare the survival outcomes across different racial and ethnic groups based on their preoperative chemosensitivity to NACT. In our study, rather than representing the preoperative chemosensitivity in a binary model as examined in prior studies (attainment of pCR vs residual disease),^8,9^ we evaluated the preoperative chemosensitivity as a spectrum, which, to our knowledge, has not been done before in breast cancer.

## Methods

### Data Source, Patient Selection, and Variables

The NCDB is a joint program from the Commission on Cancer of the American College of Surgeons and American Cancer Society and is a nationwide oncology outcomes database that collects data on approximately 70% of all new invasive cancer diagnoses in the US. In this cohort study, we included patients with early-stage breast cancer (American Joint Cancer Committee clinical stage I, II, or III disease) that was diagnosed from 2010 to 2018, were aged 18 years or older at the time of diagnosis, received NACT, and underwent curative surgery. Patients were excluded if they had metastatic disease at the time of pathologic staging, were missing clinical or pathologic staging information, or were missing race and ethnicity information. As the data are publicly available and do not contain any patient identifiers, our study was exempted from institutional review board review by Roswell Park Comprehensive Cancer Center, and informed consent was also waived for the same reason. Our study follows the Strengthening the Reporting of Observational Studies in Epidemiology (STROBE) reporting guideline.

The demographic (age, sex, race and ethnicity, insurance), clinicopathologic (clinical and pathologic TNM stage, grade, histologic characteristics of tumors), treatment (chemotherapy, hormonal therapy, surgery, and radiotherapy) characteristics, comorbidity index, and survival variables of patients were collected. Racial and ethnic information captured in the database was self-reported by patients. Race and ethnicity were self-reported as Asian, Black, Hispanic, White, or other, which includes American Indian, Aleutian or Eskimo, Hawaiian, Laotian, Pakistani, Micronesian, Chamorro/Chamoru, Guamanian, Polynesian, Tahitian, Samoan, Tongan, Melanesian, Fiji Islander, New Guinean, and Pacific Islander. The primary outcome of the study was OS, which was calculated from the date of the diagnosis until death due to any cause or last contact. Patients without a recorded death event were censored for OS at their last contact date. Preoperative chemosensitivity was defined as very sensitive (ypT0N0), sensitive (pathologic TNM stage less than clinical stage, excluding ypT0N0), and refractory (pathologic stage greater than or equal to clinical stage).

### Statistical Analysis

Data were analyzed in November 2022. The demographic, clinical, and treatment characteristics were summarized by overall sample, subtype, race and ethnicity, and chemosensitivity using median and IQR for continuous variables and frequencies and relative frequencies for categorical variables. Statistical comparisons were made using Kruskal-Wallis, Pearson χ^2^, or Fisher exact test wherever appropriate.

Multivariable Cox proportional hazards regression models were used to analyze OS as a time-to-event outcome, and controlled for age, cancer subtype, clinical stage, grade, histologic characteristics, insurance, comorbidities, chemotherapy status, hormone therapy status, radiotherapy status, time from diagnosis to chemotherapy, and time from chemotherapy to surgery. Kaplan-Meier survival curves were generated by overall sample, subtype, and race and ethnicity, and by chemosensitivity for the time-to-event outcome. The differences were examined using the log-rank test, and 3- and 5-year survival rates were recorded. Cox proportional hazards regression models were used to determine whether the association between survival and race and ethnicity differed with disease subtype or chemosensitivity cohorts. Survival was modeled as a function of race and ethnicity, subtype or chemosensitivity, and the 2-way interaction with race and ethnicity. Of interest was the overall *P* value for the interaction term. All analyses were conducted in R Studio, version 4.0.2 (R Project for Statistical Computing) with a 2-sided significance level of *P* = .05.

## Results

### Patient Characteristics and Preoperative Chemosensitivity

We identified a total of 103 605 eligible patients (median age, 53 [IQR, 44-62] years, 99.5% [n = 103 060] women, 18.2% [n = 18 888] Black patients, and 68.7% [n = 71 203] White patients) between calendar years 2010 and 2018 from the NCDB. Most patients had no comorbidities (87.1% [n = 90 243]). The baseline demographic and clinicopathologic characteristics of the cohort based on the preoperative chemosensitivity are provided in Table 1. Most patients had refractory disease (43.2% [n = 44 796]), followed by sensitive (34.4% [n = 35 638]) and very sensitive (22.4% [n = 23 171]) disease. Older patients (age >55 years) were more likely to have very sensitive disease (62.5% [n = 14 478]; *P* < .001). Among patients with very sensitive disease, 67.8% (n = 15 714) had private insurance and 27.4% (n = 6359) had government insurance compared with 61.0% (n = 27 307) with private insurance and 33.8% (n = 15 144) (*P* < .001) with government insurance in the refractory disease group. Among patients with very sensitive disease, 51.4% (n = 11 896) were node positive, while among refractory disease, 61.4% (n = 27 408) (*P* < .001) were node positive. In the hormone receptor–positive *ERBB2* negative (HR^+^) subtype, only 9.0% (n = 3837 of 42 796) were very sensitive, while among the TNBC subtype, 28.4% (n = 9224 of 32 496) were very sensitive. Among the *ERBB2*^+^ subtype, 36.7% (n = 9665 of 26 354) of the patients were very sensitive and, in both subtypes, approximately 28% to 36% were refractory compared with 58% with refractory disease among the HR^+^ subtype (eFigure 1 in Supplement 1).

**Table 1.** Baseline Characteristics Stratified Based on Preoperative Chemosensitivity^a^

Variable	Patients, No. (%)				*P* value
	Refractory (n = 44 796)	Sensitive (n = 35 638)	Very sensitive (n = 23 171)	Overall (N = 103 605)	
Age, median (IQR), y	54 (45-62)	53 (44-61)	52 (43- 60)	53 (44-62)	<.001
≤55	20 069 (44.8)	14 803 (41.5)	8693 (37.5)	43 565 (42.1)	<.001
>55	24 727 (55.2)	20 835 (58.5)	14 478 (62.5)	60 040 (57.9)	
Race and ethnicity					
Asian	1524 (3.4)	1369 (3.8)	925 (3.9)	3818 (3.7)	<.001
Black	8012 (17.9)	6673 (18.7)	4203 (18.1)	18 888 (18.2)	
. Hispanic	3463 (7.7)	2968 (8.3)	1935 (8.4)	8366 (8.1)	
White	31 252 (69.8)	24 146 (67.8)	15 805 (68.2)	71 203 (68.7)	
Other^b^	545 (1.2)	482 (1.4)	303 (1.3)	1330 (1.3)	
Sex					
Female	44 510 (99.4)	35 438 (99.4)	23 112 (99.8)	103 060 (99.5)	<.001
Male	286 (0.6)	200 (0.6)	59 (0.3)	545 (0.5)	
Insurance					
Government	15 144 (33.8)	11 573 (32.5)	6359 (27.4)	33 076 (31.9)	<.001
Private	27 307 (61.0)	22 166 (62.2)	15 714 (67.8)	65 187 (62.9)	
None	1703 (3.8)	1437 (4.0)	770 (3.3)	3910 (3.8)	
Not reported	642 (1.4)	462 (1.3)	328 (1.4)	1432 (1.4)	
Location					
Metropolitan	38 337 (87.8)	30 417 (87.6)	19 989 (88.7)	88 743 (87.9)	.001
Rural	557 (1.3)	482 (1.4)	292 (1.3)	1331 (1.3)	
Urban	4759 (10.9)	3817 (10.9)	2252 (9.9)	10 828 (10.7)	
CDCC					
0	38 560 (86.1)	31 152 (87.4)	20 531 (88.6)	90 243 (87.1)	<.001
1	5054 (11.3)	3664 (10.3)	2199 (9.5)	10 917 (10.5)	
2	902 (2.0)	619 (1.7)	328 (1.4)	1849 (1.8)	
≥3	280 (0.6)	203 (0.6)	113 (0.5)	596 (0.6)	
Clinical stage					
1	5900 (13.2)	1160 (3.6)	2864 (12.7)	9924 (9.6)	<.001
2	25 829 (57.7)	19 114 (53.6)	14 487 (62.5)	59 430 (57.4)	
3	13 067 (29.2)	15 364 (43.1)	5820 (25.1)	34 251 (33.1)	
Nodes					
Positive	27 408 (61.4)	20 955 (58.9)	11 896 (51.4)	60 259 (58.3)	<.001
Negative	17 234 (38.6)	14 581 (41.0)	11 261 (48.6)	43 076 (41.7)	
Grade					
Poor	21 479 (47.9)	21 110 (59.2)	16 744 (72.3)	59 333 (57.3)	<.001
Undifferentiated	143 (0.3)	152 (0.4)	87 (0.4)	382 (0.4)	
Moderate	16 771 (37.4)	9993 (28.0)	4030 (17.4)	30 794 (29.7)	
Well	3101 (6.9)	1520 (4.3)	314 (1.4)	4935 (4.8)	
Not reported	3302 (7.4)	2863 (8.0)	1996 (8.6)	8161 (7.9)	
ER					
Positive	28 896 (64.8)	17 829 (50.3)	6189 (26.9)	52 914 (51.4)	<.001
Negative	15 680 (35.2)	17 633 (49.7)	16 820 (73.1)	50 133 (48.7)	
PR					
Positive	24 760 (55.6)	14 408 (40.6)	4272 (18.6)	43 440 (42.2)	<.001
Negative	19 786 (44.4)	21 045 (59.7)	18 716 (81.4)	59 547 (57.8)	
*ERBB2*					
Positive	7413 (16.9)	9276 (26.5)	9665 (42.5)	26 354 (25.9)	<.001
Negative	36 542 (83.1)	25 721 (73.5)	13 072 (57.5)	75 335 (74.1)	
Subtype					
ER/PR^+^ and *ERBB2*^–^	24 967 (56.8)	13 992 (40.0)	3837 (16.9)	42 796 (42.1)	<.001
*ERBB2*^+^	7413 (16.9)	9276 (26.5)	9665 (42.5)	26 354 (25.9)	
TNBC	11 558 (26.3)	11 714 (33.5)	9224 (40.6)	32 496 (31.9)	
Time to surgery, median (IQR), d	183 (155-214)	187 (161-216)	185 (163-210)	185 (160-214)	<.001
Time to chemotherapy, median (IQR), d	32 (22-46)	31 (22-44)	29 (21-41)	31 (22-44)	<.001
Time from chemotherapy to surgery, median (IQR), d	148 (125-175)	153 (131-177)	153 (134-175)	151 (129-175)	<.001
Radiotherapy					
Yes	34 742 (77.6)	26 033 (73.1)	15 129 (65.3)	75 904 (73.3)	<.001
No	10 054 (22.4)	9605 (26.9)	8042 (34.7)	27 701 (26.7)	
Hormone therapy					
Yes	26 569 (60.3)	15 738 (45.1)	4860 (21.4)	47 167 (46.4)	<.001
No	17 496 (39.7)	19 161 (54.9)	17 886 (78.6)	54 543 (53.6)	

Abbreviations: CDCC, Charlson–Deyo Comorbidity Class; ER, estrogen receptor; *ERBB2*^–^, *ERBB2*-negative; *ERBB2*^+^, *ERBB2*-positive; PR, progesterone receptor; TNBC, triple-negative breast cancer.

^a^ Categories of chemosensitivity included very sensitive (ypT0N0), sensitive (pathologic TNM stage less than clinical stage, excluding ypT0N0), and refractory (pathologic stage greater than or equal to clinical stage).

^b^ Included American Indian, Aleutian or Eskimo, Hawaiian, Laotian, Pakistani, Micronesian, Chamorro/Chamoru, Guamanian, Polynesian, Tahitian, Samoan, Tongan, Melanesian, Fiji Islander, New Guinean, and Pacific Islander.

Baseline demographic, clinicopathologic, and treatment details stratified by race and ethnicity are given in Table 2. Among White patients, 67.0% (n = 47 740) had private insurance compared with 54.5% (n = 10 293) among Black patients and 45.9% (n = 3844) among Hispanic patients (*P* < .001). Black patients had more TNBC (42.2% [n = 7815]) and Asian patients had more *ERBB2*^+^ (33.4% [n = 1255]) (*P* < .001) compared with other races and ethnicities.

**Table 2.** Baseline Characteristics Stratified Based on Race and Ethnicity

Variable	Patients, No. (%)						*P* value
	Asian (n = 3818)	Black (n = 18 888)	Hispanic (n = 8366)	White (n = 71 203)	Other^a^ (n = 1330)	Overall (N = 103 605)	
Age, median (IQR), y	49 (41-59)	52 (44-60)	48 (41-57)	54 (45-63)	50 (42-59)	53 (44-62)	<.001
≤55	1255 (32.9)	7147 (37.8)	2447 (29.3)	32 259 (45.3)	457 (34.4)	43 565 (42.1)	<.001
>55	2563 (67.1)	11 741 (62.2)	5919 (70.8)	38 944 (54.7)	873 (65.6)	60 040 (57.9)	
Sex							
Female	3806 (99.7)	18 781 (99.4)	8331 (99.6)	70 816 (99.5)	1326 (99.7)	103 060 (99.5)	.12
Male	12 (0.3)	107 (0.6)	35 (0.4)	387 (0.5)	4 (0.3)	545 (0.5)	
Insurance							
Government	1009 (26.4)	7299 (38.6)	3201 (38.3)	21 090 (29.6)	477 (35.9)	33 076 (31.9)	<.001
Private	2555 (66.9)	10 293 (54.5)	3844 (45.9)	47 740 (67.0)	755 (56.8)	65 187 (62.9)	
None	189 (4.9)	988 (5.2)	1153 (13.8)	1519 (2.1)	61 (4.6)	3910 (3.8)	
Not reported	65 (1.7)	308 (1.6)	168 (2.0)	854 (1.2)	37 (2.8)	1432 (1.4)	
Location							
Metropolitan	3664 (98.2)	17 210 (93.1)	7914 (95.9)	58 826 (85.1)	1129 (87.3)	88 743 (87.9)	<.001
Rural	4 (0.1)	159 (0.9)	17 (0.2)	1122 (1.6)	29 (2.2)	1331 (1.3)	
Urban	64 (1.7)	1120 (6.1)	321 (3.9)	9187 (13.3)	136 (10.5)	10 828 (10.7)	
CDCC							
0	3433 (89.9)	15 578 (82.5)	7343 (87.8)	62 728 (88.1)	1161 (87.3)	90 243 (87.1)	<.001
1	339 (8.9)	2634 (13.9)	875 (10.5)	6930 (9.7)	139 (10.5)	10 917 (10.5)	
2	37 (0.9)	494 (2.6)	107 (1.3)	1193 (1.7)	18 (1.4)	1849 (1.8)	
≥3	9 (0.2)	182 (0.9)	41 (0.5)	352 (0.5)	12 (0.9)	596 (0.6)	
Clinical stage							
1	307 (8.0)	1582 (8.4)	582 (7.0)	7344 (10.3)	109 (8.2)	9924 (9.6)	<.001
2	2329 (61.0)	10 555 (55.9)	4814 (57.5)	40 937 (57.4)	795 (59.8)	59 430 (57.3)	
3	1182 (31.0)	6751 (35.7)	2970 (35.5)	22 922 (32.2)	426 (32.0)	34 251 (33.1)	
Nodes							
Positive	2186 (57.4)	11 819 (62.7)	5201 (62.4)	40 265 (56.7)	788 (59.5)	60 259 (58.3)	<.001
Negative	1623 (42.6)	7026 (37.3)	3129 (37.6)	30 761 (43.3)	537 (40.5)	43 076 (41.7)	
Grade							
Poor	2224 (58.3)	12 412 (65.7)	4830 (57.7)	39 106 (54.9)	761 (57.2)	59 333 (57.3)	<.001
Undifferentiated	18 (0.5)	80 (0.4)	31 (0.4)	248 (0.3)	5 (0.4)	382 (0.4)	
Moderate	1143 (29.9)	4413 (23.4)	2445 (29.2)	22403 (31.5)	390 (29.3)	30794 (29.7)	
Well	163 (4.3)	567 (3.0)	369 (4.4)	3776 (5.3)	60 (4.5)	4935 (4.8)	
Not reported	270 (7.1)	1416 (7.5)	691 (8.3)	5670 (8.0)	114 (8.6)	8161 (7.9)	
ER							
Positive	1998 (52.5)	7955 (42.4)	4374 (52.8)	37 869 (53.5)	718 (54.4)	52 914 (51.4)	<.001
Negative	1807 (47.5)	10 830 (57.7)	3913 (47.2)	32 981 (46.6)	602 (45.6)	50 133 (48.7)	
PR							
Positive	1642 (43.2)	6166 (32.9)	3598 (43.4)	31 435 (44.4)	599 (45.5)	43 440 (42.2)	<.001
Negative	2161 (56.8)	12 605 (67.2)	4687 (56.6)	39 376 (55.6)	718 (54.5)	59 547 (57.8)	
*ERBB2*							
Positive	1255 (33.4)	4049 (21.9)	2114 (25.9)	18 568 (26.6)	368 (28.4)	26 354 (25.9)	<.001
Negative	2506 (66.6)	14 481 (78.2)	6053 (74.1)	51 368 (73.5)	927 (71.6)	75 335 (74.1)	
Subtype							
ER/PR^+^ and *ERBB2*^–^	1549 (41.2)	6656 (35.9)	3565 (43.7)	30 462 (43.6)	564 (43.6)	42 796 (42.1)	<.001
*ERBB2*^+^	1255 (33.4)	4049 (21.9)	2114 (25.9)	18 568 (26.6)	368 (28.4)	26 354 (25.9)	
TNBC	956 (25.4)	7815 (42.2)	2484 (30.4)	20 879 (29.9)	362 (28.0)	32 496 (32.0)	
Time to surgery, median (IQR), d	187 (162-215)	194 (166-226)	199 (170-231)	182 (157-209)	190 (164-222)	185 (160-214)	<.001
Time to chemotherapy, median (IQR), d	33 (22-47)	35 (25-50)	37 (26-55)	29 (21-41)	33 (22-48)	31 (22-44)	<.001
Time from chemotherapy to surgery, median (IQR), d	152 (132-175)	154 (132-182)	158 (133-184)	149 (128-174)	154 (132-181)	151 (129-175)	<.001
Radiotherapy							
Yes	2781 (72.8)	14 198 (75.2)	6044 (72.2)	51 905 (72.9)	976 (73.4)	75 904 (73.3)	<.001
No	1037 (27.2)	4690 (24.8)	2322 (27.8)	19 298 (27.1)	354 (26.6)	27 701 (26.7)	
Hormone therapy							
Yes	1807 (48.2)	7062 (38.1)	3845 (47.0)	33 850 (48.4)	603 (46.0)	47 167 (46.4)	<.001
No	1940 (51.8)	11 466 (61.9)	4343 (53.0)	36 086 (51.6)	708 (54.0)	54 543 (53.6)	
Chemosensitivity^b^							
Refractory	1524 (39.9)	8012 (42.4)	3463 (41.4)	31 252 (43.9)	545 (410.0)	44 796 (43.2)	<.001
Sensitive	1369 (35.9)	6673 (35.3)	2968 (35.5)	24 146 (33.9)	482 (36.2)	35 638 (34.4)	
Very sensitive	925 (24.2)	4203 (22.3)	1935 (23.1)	15 805 (22.2)	303 (22.8)	23 171 (22.4)	

Abbreviations: CDCC, Charlson–Deyo Comorbidity Class; ER, estrogen receptor; *ERBB2*^–^, *ERBB2*-negative; *ERBB2*^+^, *ERBB2*-positive; PR, progesterone receptor; TNBC, triple-negative breast cancer.

^a^ Other includes American Indian, Aleutian or Eskimo, Hawaiian, Laotian, Pakistani, Micronesian, Chamorro/Chamoru, Guamanian, Polynesian, Tahitian, Samoan, Tongan, Melanesian, Fiji Islander, New Guinean, and Pacific Islander.

^b^ Categories of chemosensitivity included very sensitive (ypT0N0), sensitive (pathologic TNM stage less than clinical stage, excluding ypT0N0), and refractory (pathologic stage greater than or equal to clinical stage).

### Association of Race and Chemosensitivity

**Figure 1.**
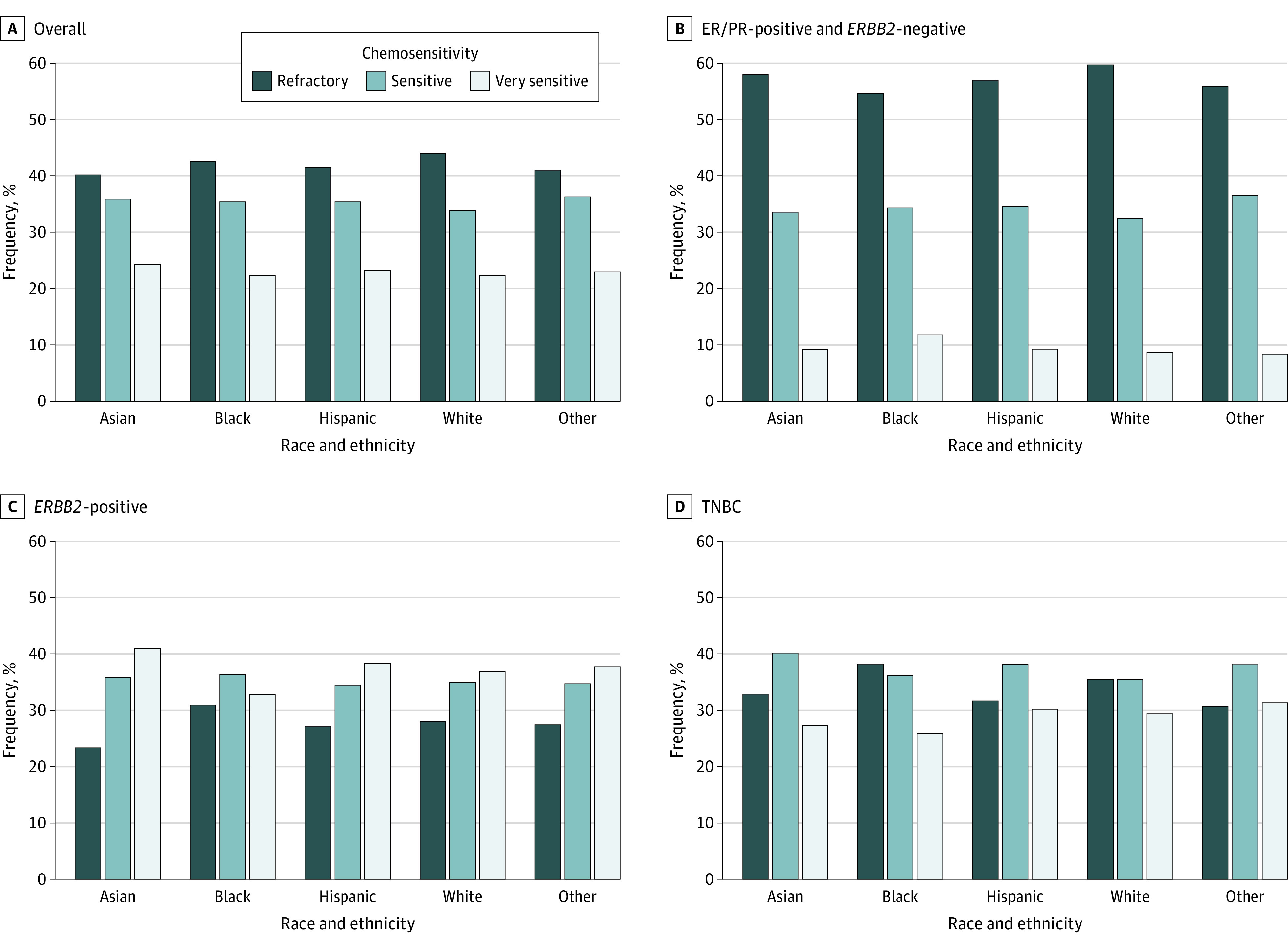
Race and Ethnicity and Preoperative Chemosensitivity Other racial and ethnic groups included American Indian, Aleutian or Eskimo, Hawaiian, Laotian, Pakistani, Micronesian, Chamorro/Chamoru, Guamanian, Polynesian, Tahitian, Samoan, Tongan, Melanesian, Fiji Islander, New Guinean, and Pacific Islander. Categories of chemosensitivity included very sensitive (ypT0N0), sensitive (pathologic TNM stage less than clinical stage, excluding ypT0N0), and refractory (pathologic stage greater than or equal to clinical stage). ER indicates estrogen receptor; PR, progesterone receptor; and TNBC, triple-negative breast cancer.

Among the HR^+^ subtype, patients had more refractory disease regardless of race and ethnicity (all races and ethnicities: 54%-59%; *P* < .001). Among those with *ERBB2*^+^ disease, Black patients had a lower percentage of very sensitive disease (32% vs 37%-40%; *P* < .001) and a higher percentage of refractory disease (16.9%; *P* < .001) compared with other races and ethnicities. Similarly, in TNBC, Black patients had more refractory disease (38.1%) than sensitive (36.1%) and very sensitive (25.7%) (*P* < .001) disease, whereas other races and ethnicities had less refractory disease (Figure 1).

### Survival Analysis

**Figure 2.**
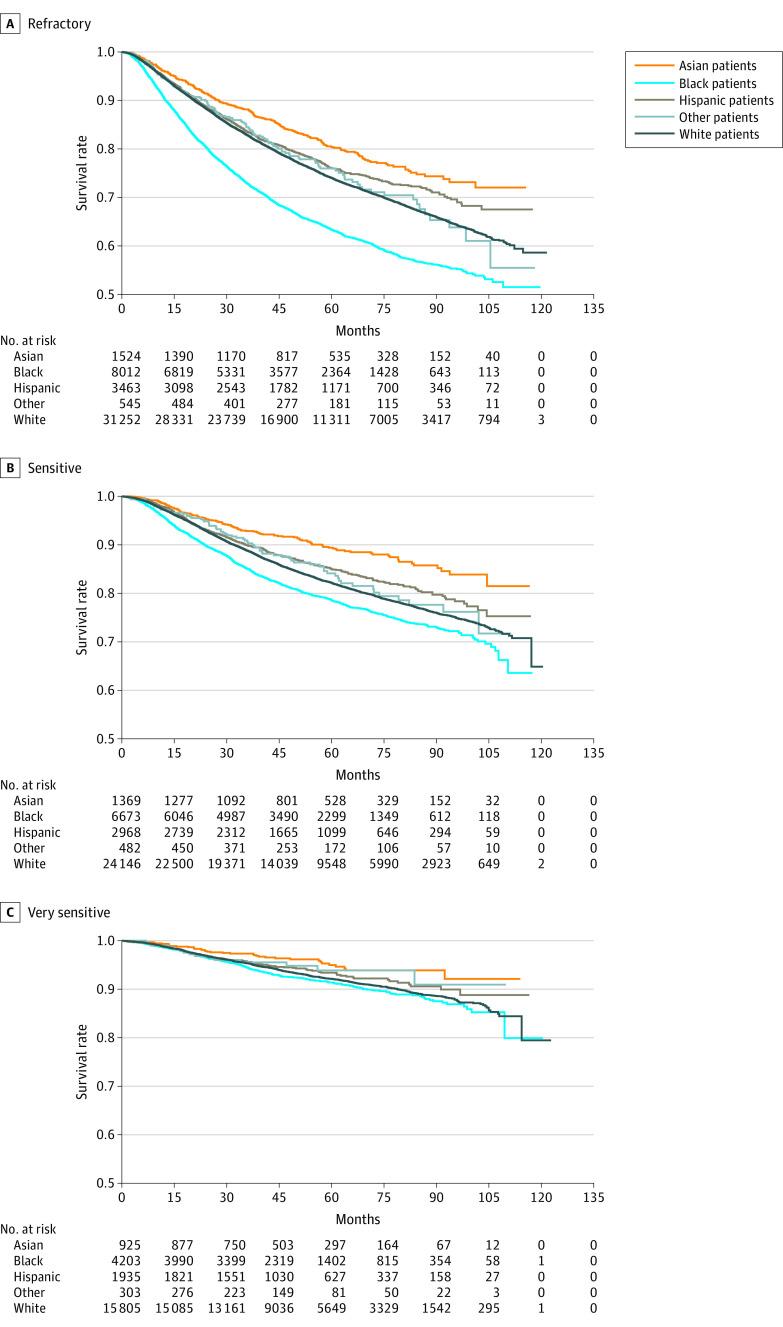
Survival by Chemosensitivity and Race and Ethnicity Other racial and ethnic groups included American Indian, Aleutian or Eskimo, Hawaiian, Laotian, Pakistani, Micronesian, Chamorro/Chamoru, Guamanian, Polynesian, Tahitian, Samoan, Tongan, Melanesian, Fiji Islander, New Guinean, and Pacific Islander. Categories of chemosensitivity included very sensitive (ypT0N0), sensitive (pathologic TNM stage less than clinical stage, excluding ypT0N0), and refractory (pathologic stage greater than or equal to clinical stage).

**Figure 3.**
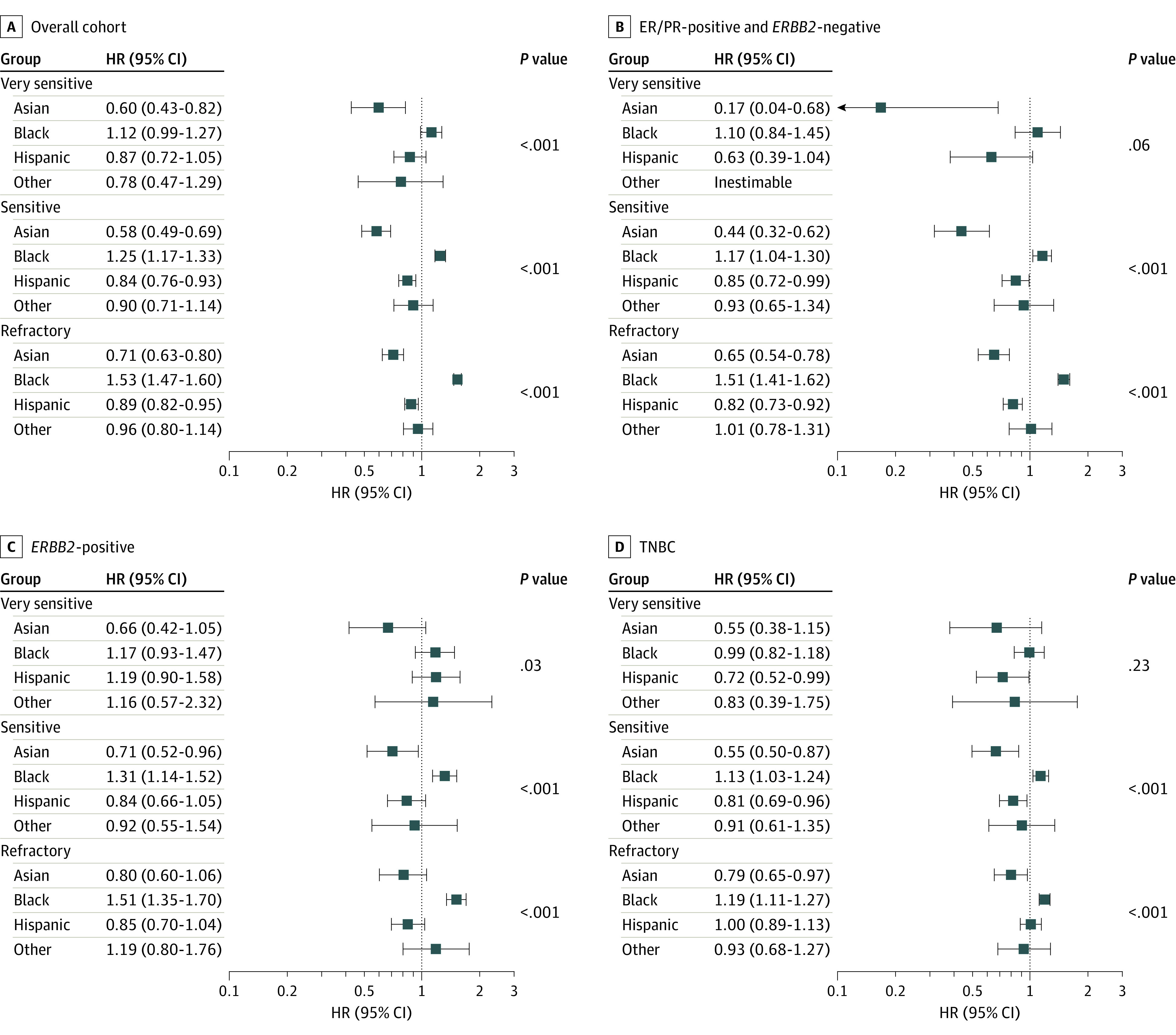
Mortality Data in Different Preoperative Sensitivity Groups Mortality data for racial and ethnic groups (White race as reference). Categories of chemosensitivity included very sensitive (ypT0N0), sensitive (pathologic TNM stage less than clinical stage, excluding ypT0N0), and refractory (pathologic stage greater than or equal to clinical stage). ER indicates estrogen receptor; HR, hazard ratio; PR, progesterone receptor; and TNBC, triple-negative breast cancer.

In the overall cohort, Black patients had significantly lower 3-year OS (Asian: refractory, 88.2%; sensitive, 92.8%; very sensitive, 97.3%; Black: refractory, 72.8%; sensitive, 85.0%; very sensitive, 94.4%; Hispanic: refractory, 83.6%; sensitive, 89.9%; very sensitive, 95.6%; White: refractory, 82.8%; sensitive, 88.8%; very sensitive, 95.3%) and 5-year OS (Asian: refractory, 80.4%; sensitive, 89.4%; very sensitive, 96%; Black: refractory, 63.4%; sensitive, 78.7%; very sensitive, 91.4%; Hispanic: refractory, 76.1%; sensitive, 85.0%; very sensitive, 93.4%; White: refractory, 74.0%; sensitive, 82.2%; very sensitive, 92.1%) (eTable 1 in Supplement 1; Figure 2) and higher mortality risk compared with all other races and ethnicities (Figure 3). This difference was more prominent in the refractory and sensitive groups. The association between survival and race and ethnicity appeared to be dependent on disease subtype (*P* < .001 for interaction) and chemosensitivity status (*P* = .003 for interaction). The racial and ethnic differences decreased with chemosensitivity (Figure 2) and were less pronounced in the TNBC cohort compared with the other subtypes. In refractory (hazard ratio [HR], 1.53; 95% CI, 1.47-1.60; *P* < .001) and sensitive (HR, 1.25; 95% CI, 1.17-1.33; *P* < .001) disease, Black patients had a higher mortality risk compared with White patients in the overall cohort. A similar observation was noted in very sensitive disease; however, it was not statistically significant (HR, 1.12; 95% CI, 0.99-1.27) (Figure 3). In the subset analysis also based on breast cancer subtypes, Black patients with refractory and sensitive disease had a higher mortality risk compared with White patients in the corresponding disease groups (refractory: HR^+^ subtype, HR, 1.51; 95% CI, 1.41-1.62; *P* < .001; *ERBB2*^+^, HR, 1.51; 95% CI, 1.35-1.70; *P* < .001; TNBC, HR, 1.19; 95% CI, 1.11-1.27; *P* < .001; sensitive: HR^+^ subtype, 1.17; 95% CI, 1.04-1.30; *P* < .001; *ERBB2*^+^, HR, 1.31; 95% CI, 1.14-1.52; *P* < .001; TNBC, HR, 1.13; 95% CI, 1.03-1.24; *P* < .001). Asian patients consistently had a lower mortality risk compared with White patients. This difference was statistically significant among refractory (HR, 0.71; 95% CI, 0.63-0.80; *P* < .001), sensitive (HR, 0.58; 95% CI, 0.49-0.69; *P* < .001), and very sensitive (HR, 0.60; 95% CI, 0.43-0.82; *P* < .001) disease in the overall cohort and HR^+^ subtype (Figure 3).

Among breast cancer subtypes, the OS for refractory (3-year, 65%; 5-year, 56%) and sensitive (3-year, 83%; 5-year, 76%) disease was significantly lower in the TNBC subtype compared with other subtypes (HR^+^ refractory: 3-year, 88%; 5-year, 79%; sensitive: 3-year, 90%; 5-year, 83%; *ERBB2*^+^ refractory: 3-year, 85%; 5-year, 77%; sensitive: 3-year, 92%; 5-year, 86%; *P* < .001) (eTable 2 in Supplement 1). The 3- and 5-year survival rates of different races and ethnicities and subtypes based on preoperative chemosensitivity are shown in Figure 2 and eFigure 2 in Supplement 1.

## Discussion

Our study found that Black patients diagnosed with TNBC and *ERBB2*^+^ breast cancer had more refractory disease to NACT, whereas those of other races and ethnicities, especially Asian patients, had more sensitive disease. Neoadjuvant chemotherapy and the attainment of pCR are associated with improved survival of patients with breast cancer.^3^ However, in our study, we found that the survival benefit of NACT differed across preoperative chemosensitivity levels and across races and ethnicities. Patients with very sensitive disease had improved survival compared with those with sensitive disease and those with refractory disease. In all subtypes, Black patients had lower 3- and 5-year OS regardless of chemosensitivity (including those who attained pCR or had very sensitive disease) compared with other races and ethnicities, although this disparity was more predominant among those with residual disease after NACT (refractory and sensitive disease groups). Black patients had a higher mortality rate in the refractory and sensitive groups compared with White patients in the overall cohort as well as within the breast cancer subtypes. It was also observed that Asian patients had higher 3- and 5-year survival rates in all chemosensitivity groups compared with other races and ethnicities and less mortality risk compared with White patients in the overall breast cancer cohort. Additionally, Hispanic patients had higher survival rates compared with White patients irrespective of chemosensitivity and breast cancer subtypes.

Since Black patients who do not attain pCR have worse survival compared with other races and ethnicities, our results highlight the need to design more effective and personalized treatment strategies for Black patients to help them attain pCR, especially in the TNBC subtype. Understanding the reasons behind the disparities that we observed in breast cancer preoperative chemosensitivity and survival among races and ethnicities is pivotal for developing personalized therapeutic options. A recent study by Bansil et al^10^ evaluating the tumor microenvironment of patients with breast cancer showed higher levels of tumor-infiltrating lymphocytes in Asian compared with White patients. Emerging evidence suggests that the tumor microenvironment is likely contributing to racial and ethnic disparities and overall survival in patients with breast cancer. Black patients exhibited an increased presence of protumorigenic immune cells, including M2 macrophages and T regulatory cells; a higher prevalence of exhausted CD8^+^ T-cell signatures; enhanced angiogenesis; neovascularization; and greater microvascular density. These factors collectively contribute to the development of aggressive tumors, tumor progression, metastasis, and, consequently, inferior disease outcomes.^11,12,13,14^

Asian and Hispanic patients were found to have better survival outcomes in our study, which agrees with the findings from other studies.^15,16,17^ Many Asian individuals have lower body mass index and fewer comorbidities, which are known to be protective mediators in breast cancer mortality.^17,18^ Asian patients are reported to have higher *ERBB2*^+^ disease, which has a good response to NACT, and may be another reason for their improved survival.^15^ Pan et al^19^ found that Asian patients with breast cancer tumors have higher immune scores and higher levels of CD8^+^ T cells, macrophages, and cytotoxic natural killer cells, which suggest improved response to immunotherapy and better survival outcomes. Other studies have noted that Black patients often experience multiple chronic comorbidities and a poorer performance status compared with other racial groups. These factors may contribute to their inferior survival rates.^20,21^ Due to the limited inclusion of racial and ethnic minority groups in clinical trials, our understanding of neoadjuvant chemotherapy in various racial and ethnic groups is restricted. A pooled analysis of National Surgical Adjuvant Breast and Bowel Project trials revealed that Black patients experienced significantly inferior distant relapse-free survival in HR^+^ localized breast cancer treated with NACT.^22^

In our study, patients belonging to different races and ethnicities responded to NACT differently in all breast cancer subtypes. However, in HR^+^ breast cancer, patients of all races and ethnicities had higher rates of refractory disease. This is in line with previous studies showing higher pCR after NACT in aggressive breast cancer subtypes, such as *ERBB2^+^* and TNBC.^23,24^ Similar to our study, the rate of pCR was higher for TNBC (68%) and lower for HR^+^ (17.4%) in the I-SPY2 trial.^25^ Molecular biology assessment of patients can identify those who may have excellent responses to NACT. With the widespread use of chemotherapy and immunotherapy as NACT in TNBC, the survival of patients has increased; however, NACT causes serious adverse effects.^8^ Therefore, rather than giving toxic chemotherapy and immunotherapy to all patients, identifying ways to de-escalate therapeutic options without affecting the efficacy by identifying the predictive biomarkers is essential.^26^ Based on our study results, race and ethnicity may be considered an important predictive marker when considering neoadjuvant therapeutic options for patients. This is especially important in light of studies showing higher pCR to neoadjuvant chemoimmunotherapy in Black compared with White patients.

Our study is a comprehensive evaluation of racial and ethnic disparities in preoperative chemosensitivity in patients with early-stage breast cancer who received NACT using a US national database. Prior studies on breast cancer describe preoperative chemosensitivity in a binary model: pCR and residual disease. Patients with residual disease may have different clinical outcomes based on their burden of residual disease. To our knowledge, this study is the first describing preoperative chemosensitivity when analyzed by 3 groups: very sensitive, sensitive, and refractory. Although the current standard is to use residual cancer burden staging, which provides further stratification for residual disease, this classification is currently unavailable in the NCDB and hence, our analysis provides more granularity into the residual disease assessment. This classification may help us to understand more about the biological behavior and clinical outcomes of patients with breast cancer with residual disease.

### Strengths and Limitations

The major strength of our study is the extensive and diverse sample size that allowed us to examine the racial and ethnic disparities in preoperative chemosensitivity in different subtypes of breast cancer with adequate statistical power; this makes our study results generalizable. In addition, the NCDB captures most hospital admissions in the US, which also helped us to evaluate the diverse patient population and treatment paradigms of breast cancer from different geographic regions.

Our study also has limitations. While pCR is a robust variable, there is a major limitation in the sensitive and refractory categories. The definition of these categories is dependent on the change between the c-stage to the yp-stage. However, in 31.8% of the cases, there is a discordance between the clinical and pathologic stage: 8.7% upstaging and 23.1% downstaging.^27^ Nevertheless, the sensitive and refractory categories help us to dissect the residual disease classification.

A limitation of the NCBD is the few treatment details, including the unavailability of specific treatment regimens, dose, duration, and lack of other clinically relevant information, such as cause of death, details of relapse and recurrence of disease, and adverse events. In addition, if patients received NACT at an institution other than the reporting institution, their chemotherapy information may be missing and might have been eliminated from the study due to missing study variables. Because this was a retrospective study, there might be other confounding factors that can influence the study results that we cannot account for. Furthermore, the NCDB does not capture information on genetic mutations, such as *BRCA1/2*, *ATM*, and *PALB2*, which could also affect the response to NACT and the survival outcomes in patients of different races and ethnicities.

## Conclusions

This cohort study found that Black patients with sensitive and refractory preoperative chemosensitivity after NACT had inferior survival, whereas Asian and Hispanic patients had better survival in comparison with White patients. Preoperative chemosensitivity may be incorporated into the decision-making process of NACT administration for patients from different racial and ethnic backgrounds. Our findings may have important implications in the design and development of future research to personalize treatment options for patients with breast cancer to improve efficacy and reduce toxic effects.

## Notes

Supplement 1. **eTable 1.** The 3-Year and 5-Year Survival Rates by Chemosensitivity and Race **eTable 2.** The 3-Year and 5-Year Survival Rates by Chemosensitivity and Subtypes **eFigure 1.** Preoperative Chemosensitivity of Stage I-III Breast Cancer **eFigure 2.** Kaplan-Meier Survival Curves by Chemosensitivity and Subtypes of Breast Cancer

Supplement 2.
Data Sharing Statement
