# Supplementary material for: Racial and Ethnic Disparity in Preoperative Chemosensitivity and Survival in Patients With Early-Stage Breast Cancer
Source: JAMA Netw Open. 2023 Nov 22;6(11):e2344517. doi: 10.1001/jamanetworkopen.2023.44517 (PMC10665980; doi:10.1001/jamanetworkopen.2023.44517)
Supplement: Supplement 1. — eTable 1. The 3-Year and 5-Year Survival Rates by Chemosensitivity and Race eTable 2. The 3-Year and 5-Year Survival Rates by Chemosensitivity and Subtypes eFigure 1. Preoperative Chemosensitivity of Stage I-III Breast Cancer eFigure 2. Kaplan-Meier Survival Curves by Chemosensitivity and Subtypes of Breast Cancer [file jamanetwopen-e2344517-s001.pdf]

Supplementary Online Content

Roy AM, Patel A, Catalfamo K, et al. Racial and ethnic disparity in preoperative chemosensitivity and survival in patients with early-stage breast cancer. *JAMA Netw Open*. 2023;6(11):e2344517. doi:10.1001/jamanetworkopen.2023.44517

eTable 1. The 3-Year and 5-Year Survival Rates by Chemosensitivity and Race

eTable 2. The 3-Year and 5-Year Survival Rates by Chemosensitivity and Subtypes

eFigure 1. Preoperative Chemosensitivity of Stage I-III Breast Cancer

eFigure 2. Kaplan-Meier Survival Curves by Chemosensitivity and Subtypes of Breast Cancer

This supplementary material has been provided by the authors to give readers additional information about their work.

© 2023 Roy AM et al. *JAMA Network Open.*

eTable 1: The 3-year and 5-year survival rates by chemosensitivity and race

Chemosensitivity	Survival Rates	Whites (%) (95% CI)	Black Patients (%) (95% CI)	Hispanics (%) (95% CI)	Asian (%) (95% CI)	P-Value
**Refractory**	3-year	82.8 (82.4, 83.3)	72.8 (71.8, 73.9)	83.6 (82.3, 94.9)	88.2 (86.5, 89.9)	**< 0.001**
	5-year	74.0 (73.5, 74.6)	63.4 (62.2, 64.6)	76.1 (74.5, 77.7)	80.38 (78.1, 82.8)	
**Sensitive**	3-year	88.8 (88.4, 89.2)	85.0 (84.1, 85.9)	89.9 (88.8, 91.0)	92.8 (91.4, 94.3)	**< 0.001**
	5-year	82.2 (81.6, 82.7)	78.7 (77.5, 79.8)	85.0 (83.5, 86.5)	89.4 (87.5, 91.3)	
**Very Sensitive**	3-year	95.3 (95.0, 95.7)	94.4 (93.7, 95.2)	95.6 (94.7, 96.6)	97.3 (96.3, 98.4)	**< 0.001**
	5-year	92.1 (91.7, 92.6)	91.4 (90.4, 92.4)	93.4 (92.1, 94.7)	95.0 (93.2, 96.8)	

CI: Confidence Interval

© 2023 Roy AM et al. *JAMA Network Open.*

eTable 2: The 3-year and 5-year survival rates by chemosensitivity and subtypes

Chemosensitivity	Survival Rates	ER/PR+ & ERBB2- (%) 95% CI)	ERBB2+ (%) (95% CI)	TNBC (%) (95% CI)	P-Value
**Refractory**	3-year	87.5 (87.1, 87.9)	85.4 (84.5, 86.2)	65.2 (64.3, 66.1)	**< 0.001**
	5-year	78.6 (78.0, 79.1)	76.9 (75.9, 78.0)	56.4 (55.4, 57.4)	
**Sensitive**	3-year	90.5 (90.0, 91.0)	91.7 (91.1, 92.3)	83.1 (82.4, 83.8)	**< 0.001**
	5-year	83.6 (82.9, 84.3)	86.3 (85.5, 87.1)	76.8 (76.0, 77.7)	
**Very Sensitive**	3-year	95.0 (94.3, 95.7)	96.5 (96.1, 96.9)	94.3 (93.8, 94.7)	**< 0.001**
	5-year	91.4 (90.3, 92.4)	93.4 (92.8, 94.0)	91.5 (90.8, 92.1)	

ER/PR+ and ERBB2-: Estrogen-receptor/progesterone receptor positive ERBB2 negative; ERBB2 +: Formerly known as human epidermal growth factor receptor 2 positive, TNBC: Triple negative breast cancer. CI: Confidence Interval

© 2023 Roy AM et al. *JAMA Network Open.*

eFigure 1: Preoperative chemosensitivity of stage I-III breast cancer.

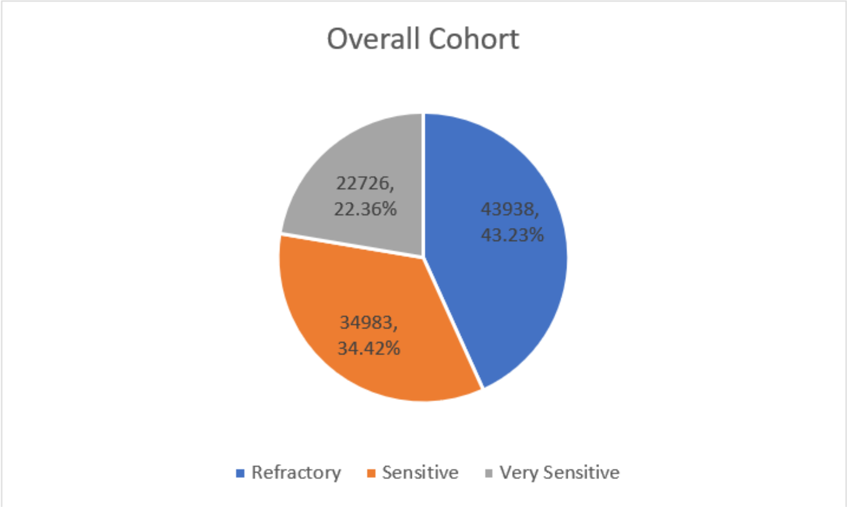

eFigure 1a: Preoperative chemosensitivity of overall cohort of stage I-III breast cancer patients. Total number =103605

© 2023 Roy AM et al. *JAMA Network Open.*

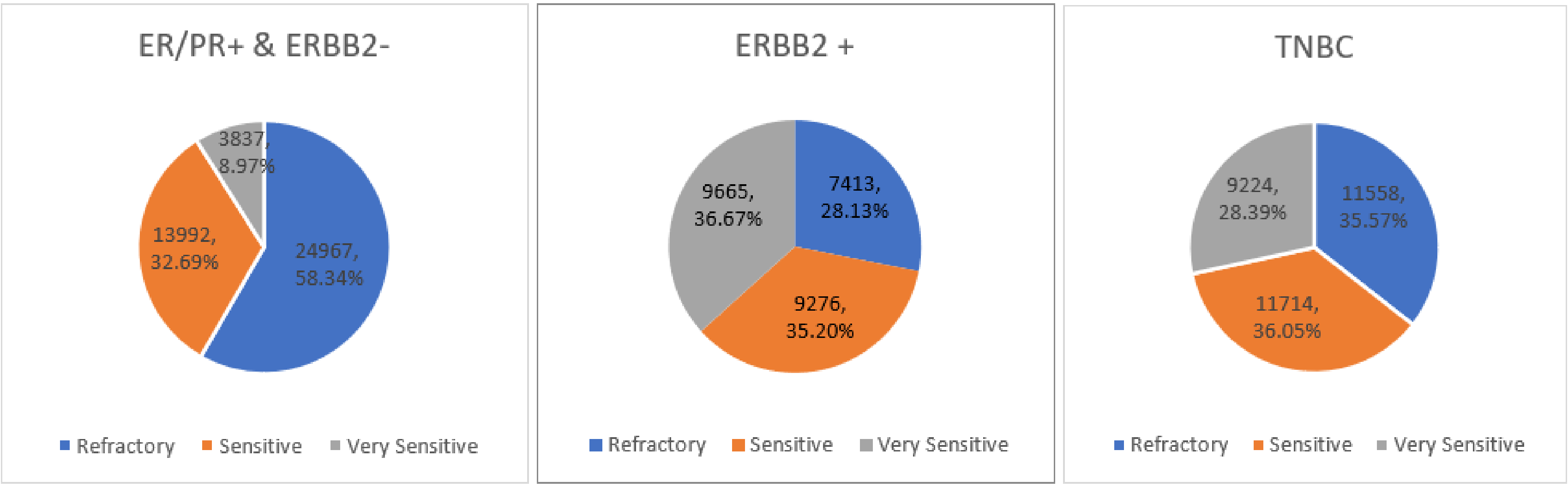

eFigure 1b: Preoperative chemosensitivity of stage I-III breast cancer patients by subtypes. Total number (n) =42796 for estrogen-receptor/progesterone receptor positive ERBB2 negative (ER/PR+ and ERBB2-), n = 26354 for ERBB2 positive (HER2 +), n = 32496 for triple negative breast cancer (TNBC).

© 2023 Roy AM et al. *JAMA Network Open.*

eFigure 2: Kaplan-Meier survival curves by chemosensitivity and subtypes of breast cancer

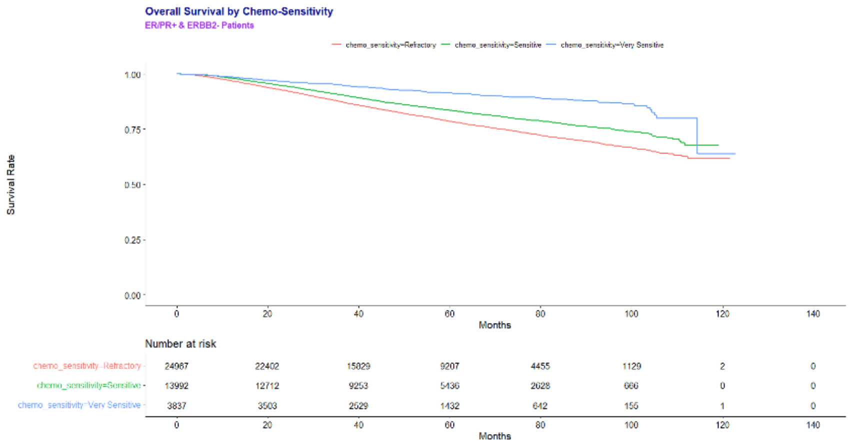

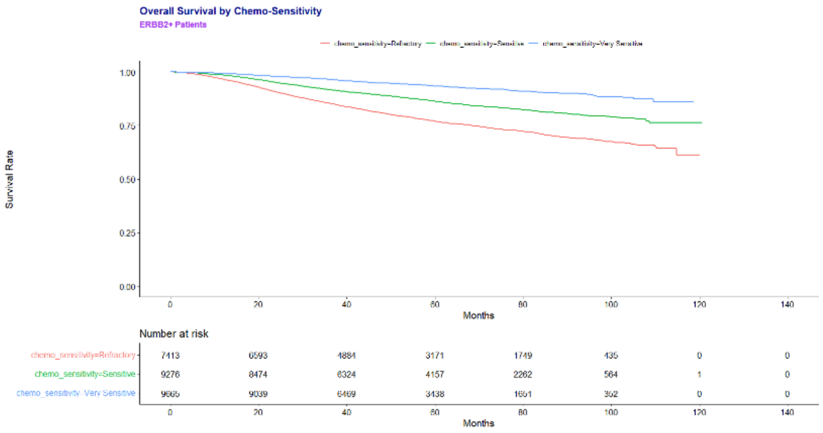

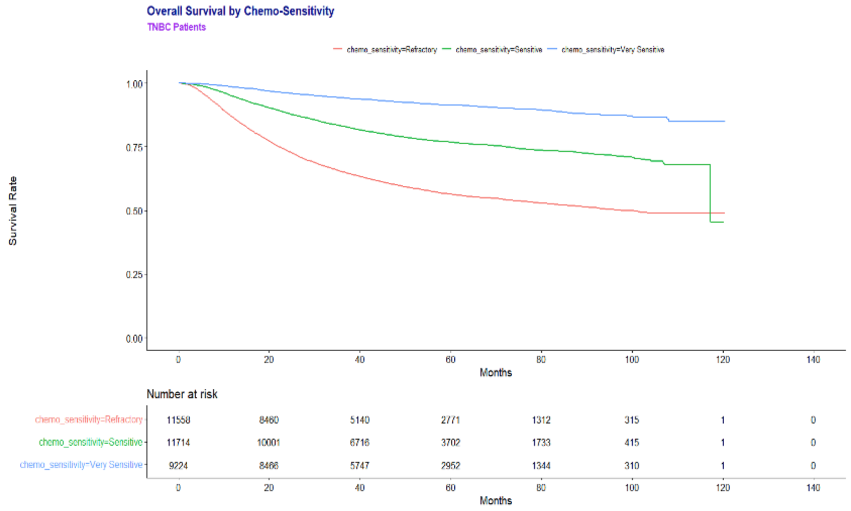

© 2023 Roy AM et al. *JAMA Network Open.*

eFigure 2: Kaplan Meir survival curves by chemosensitivity and subtypes of breast cancer. ER/PR+ and ERBB2-: Estrogen-receptor/progesterone receptor positive ERBB2 negative; ERBB2 +: Formerly known as human epidermal growth factor receptor 2 positive, TNBC: Triple negative breast cancer.

© 2023 Roy AM et al. *JAMA Network Open.*
