# Supplementary material for: Racial and Ethnic Disparity in Preoperative Chemosensitivity and Survival in Patients With Early-Stage Breast Cancer
Source: JAMA Netw Open. 2023 Nov 22;6(11):e2344517. doi: 10.1001/jamanetworkopen.2023.44517 (PMC10665980; doi:10.1001/jamanetworkopen.2023.44517)
Supplement: Supplement 2. — Data Sharing Statement [file jamanetwopen-e2344517-s002.pdf]

Data Sharing Statement

Roy. Racial and Ethnic Disparity in Preoperative Chemosensitivity and Survival in Patients With Early-Stage Breast Cancer. *JAMA Netw Open*. Published November 22, 2023. doi:10.1001/jamanetworkopen.2023.44517

Data
Data available: Yes
Data types: Deidentified participant data
How to access data: Request for data should be sent to corresponding authors: shipra.gandhi@roswellpark.org
When available: With publication

Supporting Documents
Document types: None

Additional Information
Who can access the data: Anyone requesting the data
Types of analyses: for any purpose
Mechanisms of data availability: with a signed data access agreement
